# Data for numerical solution of Caputo's and Riemann–Liouville's fractional differential equations

**DOI:** 10.1016/j.dib.2020.105375

**Published:** 2020-03-05

**Authors:** David E. Betancur-Herrera, Nicolas Muñoz-Galeano

**Affiliations:** Grupo en Manejo Eficiente de la Energía (GIMEL), Departamento de Ingeniería Eléctrica, Universidad de Antioquia (UdeA), Calle 70 No. 52-21, Medellin 050010, Colombia

**Keywords:** Fractional differential equations, Riemann–Liouville's derivatives, Caputo's derivatives, Numerical method

## Abstract

The data presented in this paper are related to the paper entitled “A Numerical Method for Solving Caputo's and Riemann-Liouville's Fractional Differential Equations which includes multi-order fractional derivatives and variable coefficients”, available in the “Communications in Nonlinear Science and Numerical Simulation” journal. Here, data are included for three of the four examples of Fractional Differential Equation (FDE) reported in [1], the other data is already available in [1]. Data for each example contain: the interval of the solution, the solution by using the proposed method, the analytic solution and the absolute error. Data were obtained through Octave 5.1.0 simulations. For a better comprehension of the data, a pseudo-code of three stages and nine steps is included.

**Table:** **Specification Table**

Subject	Numerical Analysis
Specific subject area	Numerical Solution of Fractional differential equations with Riemann-Liouville's and Caputo's Derivatives
Type of data	Table
How data were acquired	Data were acquired: Simulation. Instruments: Computer, Software Octave 5.1.0. Model and make of the instruments used: Intel i3 8^th^ generation, 8 GB Ram and 2 GB GPU.
Data format	Raw
Parameters for data collection	The data for the simulations were obtained taking into account the following parameters: 1) step size h≤0.1. 2) Fractional orders 0≤v≤3. Each point was obtained by using only one iteration.
Description of data collection	The data were obtained through simulations performed in Octave 5.1.0. Each simulation consists on taking a FDE with its respective initial conditions and parameters, and then numerically solved by using the proposed method [1]. Results of each simulation were packed in column vectors and then saved as files in plane text.
Data source location	Universidad de Antioquia Medellín/Antioquia Colombia
Data accessibility	With the article
Related research article	David E. Betancur-Herrera, Nicolas Muñoz-Galeano A Numerical Method for Solving Caputo's and Riemann-Liouville's Fractional Differential Equations which includes multi-order fractional derivatives and variable coefficients Communications in Nonlinear Science and Numerical Simulation In Press

## 1 Value of the data

 

• Data provided in this article are useful for validating algorithms that numerically solve Caputo's and Riemann–Liouville's FDE.
• Researchers can benefit from these data given that they could develop and validate new numerical methods for the solution of Caputo's and Riemann-Liouville's FDE.
• Data of this paper can be used for performing comparative analysis with other or new solution methods in terms of precision and CPU times.

## 2 Data description

The data set consists of three Microsoft Excel files whose names and content are described below:

• File example1_mod.xlsx: This file contains the solution of (1) [2]. Column A contains the values of *x* that shift from 0 to 1 in increments of 0.1. Numerical solutions of the FDE by using the proposed method are shown in columns B to E for different fractional orders (*v*_1_). Data for the numerical solution are: 1) in column B for $v_{1}=0.1$, 2) in column C for $v_{1}=0.25$, 3) in column D for $v_{1}=0.5$, and 4) in column E for $v_{1}=0.9$. Columns G to J contain the absolute error of each solution (column G for $v_{1}=0.1$, column H for $v_{1}=0.25$, column I for $v_{1}=0.5$, and column J for $v_{1}=0.9$). Finally, column L contains the analytic solution of the FDE.
  (1) $$D^{v1}f\left(x\right)+f\left(x\right)=x^{4}-\frac{1}{2}x^{3}-\frac{3}{\Gamma \left(4-v_{1}\right)}x^{3-v_{1}}+\frac{24}{\Gamma \left(5-v_{1}\right)}x^{4-v_{1}}$$
• File example2_mod.xlsx: This file contains the solution of (2). Column A contains the values of *x* that shift from 0 to 5 in increments of 0.01. Column B contains the solution of the FDE by using the proposed method. Column C contains the absolute error of the solution. Column E contains values of the analytic solution.
  (2) $$a\left(x\right)D^{v_{2}}f\left(x\right)+b\left(x\right)D^{v_{1}}f\left(x\right)+c\left(x\right)f\left(x\right)=u\left(x\right)$$

Where a(x)=x, b(x)=1, $c\left(x\right)=\sqrt{x}$ and $u\left(x\right)=\frac{2}{\Gamma (3-v_{2})}x^{3-v_{2}}-\frac{1}{\Gamma (2-v_{2})}x^{2-v_{2}}+\frac{2}{\Gamma (3-v_{1})}x^{1-v_{1}}-\frac{1}{\Gamma (2-v_{1})}x^{1-v_{1}}+\sqrt{x}\left(x^{2}-x\right)$ with $v_{1}=0.141$ and $v_{2}=0.719$.

• File example4_mod.xlsx: This file contains the solution of (3) [3]. Column A contains the values of *x* that shift from 0 to 1 in increments of 0.1. Column B contains the solution of the FDE by using the proposed method with a step size of h=0.1, while column C contains its absolute error. Column E contains the solution of the FDE by using the proposed method with a step size of h=0.01, while column F contains its absolute error. Column H contains the solution of the FDE by using the proposed method with a step size of h=0.001, while column I contains its absolute error. Column J contains values of the analytic solution.
  (3) $$aD^{2}f\left(x\right)-b{\left(x\right)}^{c}D^{v_{2}}f\left(x\right)+c\left(x\right)Df\left(x\right)+e{\left(x\right)}^{c}D^{v_{1}}f\left(x\right)+k\left(x\right)f\left(x\right)=u\left(x\right)$$

Where a=1, $b\left(x\right)=x^{\frac{1}{2}}$, $c\left(x\right)=x^{\frac{1}{3}}$, $e\left(x\right)=x^{\frac{1}{4}}$, $k\left(x\right)=x^{\frac{1}{5}}$, $u\left(x\right)=-a-\frac{b(x)}{\Gamma (3-v_{2})}x^{2-v_{2}}-c\left(x\right)x-\frac{e(x)}{\Gamma (3-v_{1})}x^{2-v_{1}}+k\left(x\right)\left(2-\frac{1}{2}x^{2}\right)$ with $v_{1}=0.333$ and $v_{2}=1.234$.

## 3 Experimental design, materials, and methods

Data were obtained after the application of the proposed method. The proposed method can be programmed by using the following pseudo code of three stages and nine steps:

• Stage 1: Entry of initial conditions and simulation parameters. In the case of having a Riemann-Liouville FDE, initial condition must be fractional. Otherwise; in the case of having a Caputo FDE, initial condition must be integer. The simulation parameters are: interval of solution, step size and fractional order. The following are the steps corresponding to this stage:
  1. Start.
  2. Initialize $x_{a}=0$, $x_{b}=b$ and l=1.
  3. Introduce simulation parameters: *h, v_j_*, with j=0,1,…,m.
  4. Introduce initial conditions$\;(G^{1}\left(0\right),\ldots ,$ $\;G^{j}\left(0\right),\ldots ,$ *G*^m^(0), $\alpha^{1-r_{j}}\left(0\right),$ $\ldots ,\alpha^{k_{J}-r_{j}}\left(0\right)$, $,\ldots ,\;\alpha^{n_{j}-r_{j}}\left(0\right),\ldots ,\;\alpha^{n_{m}-r_{j}}\left(0\right))$ for Riemann-Liouville FDE or (*f*(0), *β*^1^(0), $,\ldots ,\;\beta^{k_{j}}\left(0\right),\ldots ,\beta^{n_{m}-1}\left(0\right))$ for Caputo's FDE.
• Stage 2: First iteration. This stage is composed of the step five and is used to obtain the additional initial conditions required to solve the FDE:
  5. Calculate the fractional additional initial conditions (*G*^1^(*h*),…, $G^{j}\left(h\right),\ldots ,G^{m}\left(h\right)$) using equations of theorem 3.2 of [1] for Riemann-Liouville's FDE or calculate the integer additional initial conditions ($\beta_{0}^{n_{m}}$ $,\ldots ,\beta_{1}^{n_{m}}$) using equations of corollary 3.2.1 of [1] for Caputo's FDE.
• Stage 3: Calculation of the solution. A routine can be used to calculate each point of the solution into the interval [*x_a_*,  *x_b_*], each point is only calculated once. Steps of this stage are shown below:
  6. While $x\leq (x_{b}-h)$.
  7. Calculate $f_{l+1}$ using equations of propositions 3.1 and 3.3 of [1] for RLFDE or propositions 3.2 and 3.4 of [1] for CFDE.
  8. Calculate x=(l+1)h.
  9. End.

## Conflict of Interest

The authors declare that they have no known competing financial interests or personal relationships that could have appeared to influence the work reported in this paper.

## Appendix Supplementary materials

Image, application 1

Image, application 2

Image, application 3
